# Medical School at Buffalo

**Published:** 1846-09

**Authors:** 


### EDITORIAL DEPARTMENT.

*Medical School at Buffalo.*—Many of our readers are aware that the establishment of a medical school at Buffalo has been for some years in contemplation. The attention of members of the profession, not only in this immediate neighborhood, but at a distance, has frequently been directed to this place as a point affording peculiar advantages for medical instruction. Buffalo has now a population exceeding 30,000. An increase, in the ratio of its present progress, will more than double its present population in the space of ten years. There is every reason to suppose that the elements of growth and prosperity will develop themselves more and more, and that it will maintain its present position as the commercial emporium of the North West. Taking into view its present size, its prospective importance, and the proportionate facilities and materials for medical instruction; the extensive territory with which it is connected by means of the various channels of communication concentrating here from every direction, as to a a central point; and, it may be added, the expenses of living for the student, which are not much, if any, greater than in most of our larger villages; we think we may say without invidiousness, that if there be any place between the eastern boundary of our state and the farthest extremity of the lakes, which possesses peculiar claims of locality for a school of medicine, it is this. We are free to say that, apart from these considerations, the establishment of a medical school at the present time, might perhaps with propriety be deemed uncalled for, if not injudicious. In saying this, however, we do not intend to concede that the objections to the multiplication of medical schools, which are of late so frequently urged, are well founded. We have yet to learn that medical education has suffered, or will suffer, from free, honorable competition; and that as an exception to a maxim which is invariable in its application to every thing else, a medical school will be more efficient the more it is suffered to assume the character of a monopoly! But on this subject we purpose to submit some remarks at another time.

Formal steps preparatory to an application to the Legislature for a medical school in this place were taken about a year ago. It was then suggested by several citizens interested in the organization of an academic institution, that application should be made for a university charter, embracing all departments, and providing for their organization successively, as should be deemed expedient. The Legislature at its last session granted such a charter, and the medical department has been fully organized by establishing seven professorships, which, with the appointments by the Council of the University, are as follows: Chemistry and Pharmacy, James Hadley, M. D.; Physiology and Medical Jurisprudence, Charles B. Coventry, M. D.; General and Special Anatomy, James Webster, M. D.; Pathology and Materia Medica, Charles A. Lee, M. D.; Principles and Practice of Surgery and Clinical Surgery, Frank H. Hamilton, M. D.; Obstetrics and Diseases of Women and Children, James P. White, M. D.; Principles and Practice of Medicine and Clinical Medicine, Austin Flint, M. D. It will be observed that five of the seven chairs are occupied by incumbents of professorships in Geneva Medical College. The connection of these gentlemen with the latter institution still continues; and hence, for the mutual convenience of themselves and students who desire to attend lectures both at Geneva and Buffalo, it has been thought best that the first course at Buffalo shall be a spring course. It will commence in the latter part of February, 1847, and continue the usual period, the lectures in every department being as full as at Geneva.

We are happy to state to those who have encouraged this undertaking, and those who are inclined to regard it with interest, that the institution commences its existence under promising auspices. In so far as the active co-operation of the citizens of Buffalo is concerned, we may anticipate for it all that could be reasonably desired; and already it has received an earnest of what may be expected. Of the major portion of the faculty, well known from their labors in a neighboring institution, it is needless to speak, except to say that they will bring to the discharge of their duties all their zeal and efficiency. We assume, also, the responsibility of saying in behalf of the faculty, that it is their earnest desire, and their determination, in the management of this institution, to adopt and carry out such principles as shall best subserve the ends of medical education, and secure the confidence and approbation of the medical profession.

